# ADAM33 Silencing Inhibits Vascular Smooth Muscle Cell Migration and Regulates Cytokine Secretion in Airway Vascular Remodeling via the PI3K/AKT/mTOR Pathway

**DOI:** 10.1155/2022/8437348

**Published:** 2022-08-31

**Authors:** Fang Yan, Xin Hu, Long He, Kegang Jiao, Yanyan Hao, Jing Wang

**Affiliations:** ^1^School of Public Health, Xinjiang Medical University, Urumqi 830011, China; ^2^State Key Laboratory of Pathogenesis, Prevention and Treatment of High Incidence Diseases in Central Asia, Department of Respiratory Medicine, The First Affiliated Hospital of Xinjiang Medical University, Urumqi 830054, China; ^3^Department of Cardiology, The First Affiliated Hospital of Xinjiang Medical University, Urumqi 830054, China; ^4^Department of Respiratory Medicine, The Second Affiliated Hospital of Hainan Medical University, Haikou, Hainan 570311, China

## Abstract

**Introduction:**

Vascular smooth muscle cells (VSMCs) are highly involved in airway vascular remodeling in asthma.

**Objectives:**

This study aimed to investigate the mechanisms underlying the effects of a disintegrin and metalloproteinase-33 (ADAM33) gene on the migration capacity and inflammatory cytokine secretion of VSMCs.

**Methods:**

Human aortic smooth muscle cells (HASMCs) were transfected with lentiviral vectors carrying short hairpin RNA (shRNA) targeting ADAM33 or negative control vectors. The migration capacity of HASMCs was evaluated by a transwell assay. The levels of secreted inflammatory cytokines were measured using enzyme-linked immunosorbent assay (ELISA) kits. Reverse transcription-quantitative polymerase chain reaction and Western blot assays were performed to detect mRNA and protein expression levels.

**Results:**

Silencing of ADAM33 significantly inhibited the migration of HASMCs. The expression of tumor necrosis factor alpha (TNF-*α*) in the supernatant of HASMCs was decreased, while that of interferon gamma (IFN-*γ*) was increased after the transfection of shRNA targeting ADAM33. Insufficient ADAM33 expression also suppressed the expression levels of phosphatidylinositol 3-kinase (PI3K), phospho-protein kinase B (AKT), phospho-mammalian target of rapamycin (mTOR), Rho-associated protein kinases, phospho-forkhead box protein O1 (FOXO1), and cyclin D1, but it did not affect the levels of AKT, mTOR, or Rho.

**Conclusion:**

Silencing of the ADAM33 gene inhibited HASMC migration and regulated inflammatory cytokine secretion via targeting the PI3K/AKT/mTOR pathway and its downstream signaling. These data contribute to a better understanding of the regulatory mechanisms of airway vascular remodeling in asthma.

## 1. Introduction

Bronchial asthma is a chronic respiratory disease characterized by bronchial inflammation, airway hyper-responsiveness, and airway remodeling [1]. Morphological changes in the airway epithelium, smooth muscle, basement membrane, and blood vessels are the main manifestations of airway remodeling [2]. Previous evidence has shown that the number of capillaries in the bronchial submucosa increased by 46% in patients with severe asthma compared with healthy subjects, suggesting that angiogenesis is closely related to the severity of asthma and airway remodeling [3]. Vascular smooth muscle cells (VSMCs) play an essential role in maintaining the structure and regulating the function of blood vessels. The proliferation, apoptosis, cell cycle progression, and migration of VSMCs, as well as their ability to synthesize and secrete cytokines and extracellular matrix, can change significantly in response to pathological stimuli [4]. Biological and functional alterations in VSMCs are highly implicated in the regeneration of branchial vessels and airway remodeling [5]. However, the molecular mechanisms underlying the phenotypic transformation of VSMCs during airway vascular remodeling are not fully understood.

A disintegrin and metalloproteinase-33 (ADAM33) gene is located at chromosome 20p13 and encodes a metalloproteinase [6]. It is involved in a multitude of biological processes, including proteolysis, molecular modification, interaction between cells and matrix, and intracellular signal transduction. Previous studies have identified ADAM33 as a susceptibility gene for bronchial asthma [7]. In addition, the pulmonary expression of ADAM33 is related to the severity of asthma and airway remodeling [8]. Moreover, the ADAM33 gene contains a metalloprotease region, which has been reported to promote angiogenesis [9]. The *in vitro* culture of human embryonic lung tissues also has shown that soluble ADAM33 accelerates vascular endothelial differentiation and promotes angiogenesis [10]. Importantly, our previous study has demonstrated that the silencing of the ADAM33 gene in human aortic smooth muscle cells (HASMCs) inhibits cell proliferation and promotes apoptosis [11]. However, whether ADAM33 can affect the migration and inflammatory cytokine secretion of VSMCs during airway vascular remodeling remains unknown.

The phosphatidylinositol 3-kinase (PI3K)/protein kinase B (AKT)/mammalian target of rapamycin (mTOR) pathway regulates cell growth, proliferation, and differentiation by phosphorylating downstream transcription factors [12]. Additionally, activation of PI3K/AKT/mTOR signaling promotes cell cycle progression and induces DNA synthesis in different pathological conditions, such as cancers and pulmonary vascular diseases [13]. Our previous findings have suggested that ADAM33 may regulate HASMC proliferation and cell cycle progression via PI3K/AKT signaling [11]. Therefore, in the current study, we investigated the effects of ADAM33 silencing on HASMC migration and inflammatory cytokine secretion. The regulatory effects of ADAM33 knockout on the PI3K/AKT/mTOR pathway and its downstream signaling were also explored.

## 2. Materials and Methods

### 2.1. Reagents

Lentiviral vectors carrying short hairpin RNA (shRNA) targeting ADAM33 (LV-ADAM33) and negative control vectors were obtained from Shanghai HanBio Technology (China). The infection enhancers HitransG A and HitransG P were obtained from Jikai Gene Company (China). Enzyme-linked immunosorbent assay (ELISA) kits for interferon gamma (IFN-*γ*), tumor necrosis factor alpha (TNF-*α*), and interleukin 17 (IL-17) were purchased from Hangzhou Kelian Biological Company (China). Anti-PI3 kinase p85 alpha, antirhodopsin [1D4], and anticyclin D1-C-terminal [EPR2241] antibodies were obtained from Abcam (UK). Anti-AKT (pan) (C67E7), antiphospho-AKT (Ser473), anti-mTOR (7C10), antiphospho-mTOR (Ser2448), anti-ROCK1 (C8F7), and antiphospho-forkhead box protein (FOXO1) (C29H4) antibodies were purchased from Cell Signaling Technology Corporation (USA).

### 2.2. Cell Culture

HASMCs were obtained from Wuhan Punosel Life Sciences (China) and cultivated in complete medium (cat no.: CM-H081, Procell Life Science and Technology, Wuhan, China) with 5% CO_2_ and saturated humidity at 37°C. After passaging for 3-6 generations, immunofluorescence staining was performed to detect alpha-smooth muscle actin. Smooth muscle cells are characterized by the presence of alpha-smooth muscle actin in the cytoskeleton and cytoplasm. When HASMCs reached a confluence of ∼90%, the logarithmic growth phase of this cell line was determined by drawing a growth curve. The expression of ADAM33 in HASMCs was measured by reverse transcription–quantitative polymerase chain reaction (qRT-PCR) and Western blot assays.

### 2.3. Lentiviral Vector Construction and Cell Transfection

Our previous study showed that a lentiviral vector carrying shRNA targeting ADAM33 (LV-ADAM33) effectively silenced the ADAM33 gene in HASMCs [11]. In this study, HASMCs were transfected with LV-ADAM33 (MOI = 10, 1 × 10^7^ TU/mL; the LV-ADAM33 group) or a negative control vector (MOI = 10, 1 × 10^7^ TU/mL; the LV-NC group) for 72 h using the HitransG P reagent (cat no. REVG005, GeneChem, Shanghai, China). The transfection efficiency was evaluated by Western blot and qRT-PCR assays. The control group remained untreated.

### 2.4. Transwell Migration Assay

The bottom chamber of a transwell plate was filled with 600 *μ*L of complete medium. HASMCs from the different groups were trypsinized, and 100 *μ*L of the cell suspension (5 × 10^5^ cells/mL) was added to the upper chamber of the plate. After a 72-h incubation at 37°C, the cells were fixed with 4% formaldehyde for 20 min at room temperature, rinsed with phosphate-buffered saline (PBS), and then stained with 400 *μ*L of Giemsa solution A (cat no. D010, Nanjing Jiancheng Bioengineering Institute, Nanjing, China) at room temperature for 1 min. Then, cells were stained with 800 *μ*L of Giemsa solution B for 6 min. After washing with PBS, the cells on the surface of the upper chamber were wiped off. The chamber was then dried and moved to a glass slide. The cells were counted under a microscope in five random fields of view.

### 2.5. Measurement of Inflammatory Cytokine Levels

HASMCs were trypsinized, resuspended in complete medium, and then inoculated into a 24-well plate (500 *μ*L/well, 2.5 × 10^4^ cells/well). After a 24-h incubation at 37°C/5% CO_2_, the cells were transfected with lentiviral vectors or remained untreated, as described previously. Then the cell supernatant was collected, and the contents of IFN-*γ*, TNF-*α*, and IL-17 were measured using ELISA kits, according to the manufacturer's instructions.

### 2.6. qRT-PCR

Total RNA was extracted from cells using the Trizol method. cDNA was synthesized using the 5X All-In-One RT MasterMix with AccuRT Genomic DNA Removal Kit (cat no. G492, Applied Biological Materials Inc., Richmond, Canada). The relative mRNA expression levels of ADAM33, *PI3K*, *mTOR*, *AKT*, *Rho*, *ROCK*, *FOXO1*, and *cyclinD1* were measured by qRT-PCR using SYBR Premix Ex Taq (Takara Bio, Japan) and the Bio-Rad CFX96 Detection System (Bio-Rad Laboratories, USA). The cycling conditions were as follows: 95°C for 2 min (predenaturation), and 40 cycles of 95°C for 0.5 min (denaturation) and 60°C for 0.5 min (annealing/extension). Quantitative results were obtained using the 2-∆∆Cq method, and gene expression was normalized to that of *β*-actin. The primers used for qRT-PCR are shown in Table 1.

### 2.7. Western Blot

Cells were washed with sterile PBS (5 mL), collected, and mixed with 100 *μ*L of radioimmunoprecipitation assay buffer (cat no. AR0105, Boster Biological Technology, Wuhan, China). The protein concentration was measured by the bicinchoninic acid method. Samples were separated by 10% SDS-PAGE and then transferred to a polyvinylidene fluoride membrane with a current of 250 mA. Blocking solution containing 5% skim milk powder was used to seal the transfer membrane for 1 h. Primary antibodies (PI3K, p-AKT, AKT, p-mTOR, mTOR, Rho, ROCK, p-FOXO1, FOXO1, and cyclin D1) were diluted in tris-buffered saline containing Tween-20 (TBST). After rinsing with TBST three times (5 min each), the membrane was incubated with the primary antibody overnight at 4°C, respectively, followed by a 2-h incubation with the prediluted secondary antibody (Abcam, UK) for 2 h at room temperature. After washing with TBST three times, the membrane was analyzed using the ChemiScope Mini Chemiluminescence Instrument (Model 3000, Clinx Science Instruments Co. Ltd., Shanghai, China).

### 2.8. Statistical Analysis

Statistical analysis was performed using SPSS19.0 software. Data were expressed as the mean ± standard deviation. One-way analysis of variance was performed to compare normally distributed data with equal variance among multiple groups, while the *t*-test was used for the comparison between two groups. Data that did not conform to normality were logarithmically transformed before comparison. The Tamhane multiple comparison test was used to compare data with unequal variance. A *P*-value of <0.05 indicated a statistically significant difference.

## 3. Results and Discussion

### 3.1. Silencing of ADAM33 Suppressed the Migration Capacity of HASMCs

**Figure 1:**
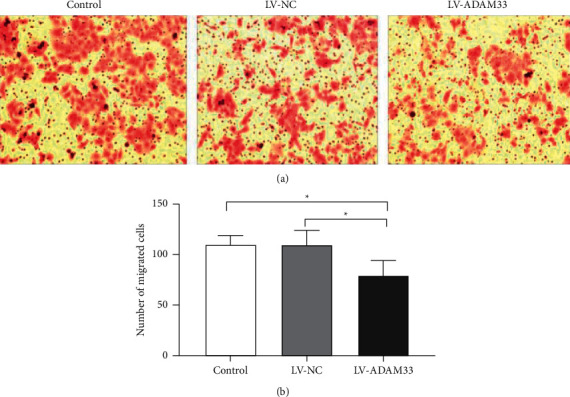
Effect of ADAM33 silencing on the migration ability of HASMCs. HASMCs were transfected with a lentiviral vector carrying shRNA targeting ADAM33 (the LV-ADAM33 group) or a negative control vector (the LV-NC group). The control group remained untreated. A transwell migration assay was performed to evaluate the migration ability of HASMCs. (a) Representative microscopic images and (b) quantitative measurement of the number of migrated cells are shown; ^*∗*^*P* < 0.05.

To explore the effects of ADAM33 silencing on HASMCs, we first assessed the migration ability of HASMCs following lentiviral transfection. Microscopic analysis showed that the HASMCs in the control and LV-NC groups were densely distributed in the bottom chamber of the plate, while the migrated cells in the LV-ADAM33 group were sparse (Figure 1(a)). Quantitative measurement revealed that the numbers of HASMCs that migrated to the lower chamber in the control, LV-NC, and LV-ADAM33 groups were 110.200 ± 8.736, 109.600 ± 14.406, and 79.400 ± 14.628, respectively. The number of migrated cells in the LV-ADAM33 group was significantly less than that of the control and LV-NC groups, while no significant difference between the control and LV-NC groups was observed (Figure 1(b)). These data suggest that the migration ability of HASMCs decreased after ADAM33 silencing.

### 3.2. Knockout of ADAM33 Regulated the Secretion of Inflammatory Cytokines

**Figure 2:**
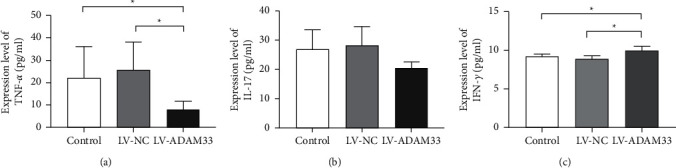
Effect of ADAM33 knockout on inflammatory cytokine production. HASMCs were transfected with LV-ADAM33 or a negative control vector (LV-NC). The control group remained untreated. The levels of (a) TNF-*α*, (b) IL-17, and (c) IFN-*γ* in the cell supernatant were measured using ELISA; ^*∗*^*P* < 0.05.

Next, the levels of inflammatory cytokines in the HASMC supernatant were assessed. The concentrations of TNF-*α* in the control, LV-NC, and LV-ADAM33 groups were 22.213 ± 13.870, 25.676 ± 12.430, and 8.045 ± 3.636 pg/mL, respectively. Silencing of ADAM33 significantly inhibited the secretion of TNF-*α* (Figure 2(a)). There was no significant difference in the level of IL-17 among these groups (Figure 2(b)). The concentrations of IFN-*γ* in the control, LV-NC, and LV-ADAM33 groups were 9.022 ± 0.284, 8.900 ± 0.410, and 9.968 ± 0.549 pg/mL, respectively. Insufficient ADAM33 expression significantly promoted the production of IFN-*γ* in HASMCs (*P* < 0.05; Figure 2(c)). The above data indicate that ADAM33 silencing inhibited the secretion of TNF-*α* but induced the production of IFN-*γ* in HASMCs.

### 3.3. ADAM33 Gene Silencing Mediated the PI3K/AKT/mTOR and Rho/ROCK Signaling Pathways

**Figure 3:**
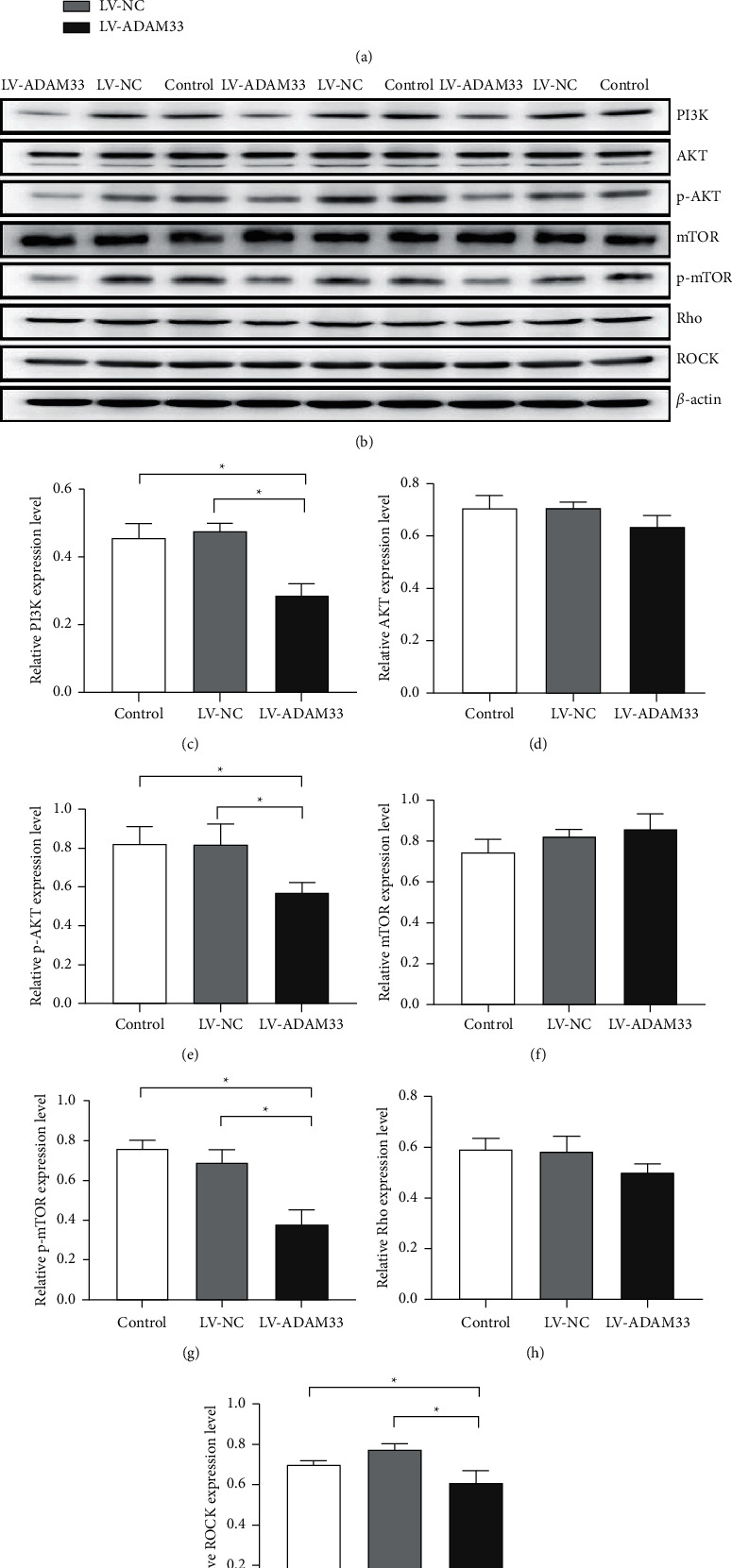
Effect of ADAM33 silencing on the PI3K/AKT/mTOR and Rho/ROCK pathways. HASMCs were transfected with LV-ADAM33 or a negative control vector (LV-NC). The control group remained untreated. (a) The mRNA expression levels of PI3K, AKT, mTOR, Rho, and ROCK were detected by qRT-PCR. (b–i) The protein levels of PI3K, AKT, p-AKT, mTOR, p-mTOR, Rho, and ROCK were measured by Western blot assay; ^*∗*^*P* < 0.05.

We further explored the effects of ADAM33 knockout on the PI3K/AKT/mTOR and Rho/ROCK pathways. The qRT-PCR results showed that the mRNA levels of PI3K and Rho in the LV-ADAM33 group were significantly lower than those of the control and LV-NC groups, while no significant difference was found in the expression levels of AKT, mTOR, and ROCK among these groups (Figure 3(a)). The protein expression of PI3K, p-AKT, p-mTOR, and ROCK in the LV-ADAM33 group was significantly downregulated compared with that of the control and LV-NC groups (Figures 3(b), 3(c), 3(e), 3(g), 3(i)), whereas the levels of AKT, mTOR, and Rho did not change significantly (Figures 3(b), 3(b),3(f), and 3(h)). The above findings imply that ADAM33 silencing suppressed the activation of PI3K/AKT/mTOR signaling and its downstream Rho/ROCK pathway.

### 3.4. Silencing of ADAM33 Downregulated the Expression of FOXO1 and Cyclin D1

**Figure 4:**
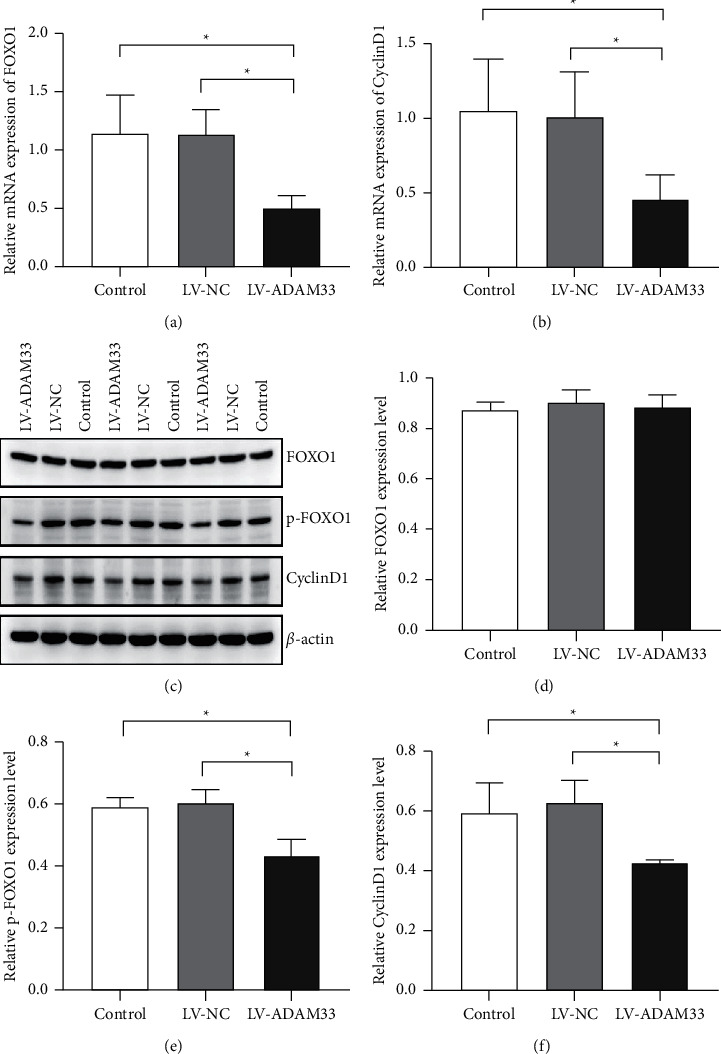
Effect of ADAM33 knockout on the expression of FOXO1 and cyclin D1. HASMCs were transfected with LV-ADAM33 or a negative control vector (LV-NC). The control group remained untreated. (a, b) The mRNA expression levels of FOXO1 and cyclin D1 were detected by qRT-PCR. (c–f) The protein levels of FOXO1, p-FOXO1, and cyclin D1 were measured by Western blot assay; ^*∗*^*P* < 0.05.

Our previous data showed that the silencing of the ADAM33 gene inhibited the cell cycle transition of HASMCs from the G0/G1 phase to the S phase, prolonged cell cycle progression, and suppressed cell proliferation [11]. To clarify the mechanism by which ADAM33 silencing regulated the cell cycle transition of HASMCs, we examined the expression levels of the downstream factors of the PI3K/AKT/mTOR pathway (the transcription factor FOXO1 and the cell cycle regulator cyclin D1) in HASMCs. In comparison with the control and LV-NC groups, the LV-ADAM33 group showed significantly decreased mRNA levels of FOXO1 and cyclin D1 (Figure 4(a), 4(b)). In addition, knockout of ADAM33 significantly downregulated the protein expression levels of p-FOXO1 and cyclin D1 (Figures 4(c)–4(f)). Taken together, ADAM33 silencing may inhibit cell cycle progression via downregulating the downstream factors of the PI3K/AKT/mTOR signaling pathway.

Previous research has mainly focused on the roles of airway epithelial cells, smooth muscle cells, and fibroblasts in asthma airway remodeling. Recent studies suggest that aberrantly expressed factors related to airway remodeling may also be used as potential therapeutic targets for asthma [14]. In the present study, we silenced the ADAM33 gene in HASMCs using lentiviral transfection and found that ADAM33 silencing reduced the migration ability and altered the inflammatory cytokine secretion of HASMCs. Further analysis revealed that the regulation of HASMCs by ADAM33 silencing probably occurred through PI3K/AKT/mTOR signaling and its downstream Rho/ROCK pathway. These findings indicate that the abnormal expression of ADAM33 may contribute to the progression of airway remodeling by changing the biological behavior of VSMCs.

ADAM33 is predominantly expressed in airway structural cells, such as smooth muscle cells, myofibroblasts, and fibroblasts [15]. The alteration in ADAM33 expression has been shown to significantly affect the development of airway remodeling. A recent study has reported that tumor growth factor beta promotes the mesenchymal transition of airway epithelial cells by increasing ADAM33 expression in them [16]. In addition, Li et al. have demonstrated that IFN-*γ* downregulates ADAM33 in human embryonic lung MRC-5 fibroblasts sensitized with *Dermatophagoides farinae* 1, causing the decreased proliferation of fibroblasts and reduced airway remodeling [17]. Moreover, Duan et al. have shown that the overexpression of soluble ADAM33 promotes the hypercontractile phenotype transition of airway smooth muscle cells (ASMCs) through the Rho/ROCK pathway and subsequently contributes to airway hyper-responsiveness in rats [18]. Also, Zhou et al. have reported that the silencing of ADAM33 decreased the proliferation and increased the apoptosis of ASMCs in a rat model of ovalbumin-induced asthma [19]. The phenotypic transformation of VSMCs, such as migration, proliferation, apoptosis, and secretion of extracellular matrix, is a hallmark of airway vascular remodeling [20]. Our previous findings and the present study showed that silencing the ADAM33 gene not only inhibited the cell cycle progression of HASMCs but also reduced their migration capacity. It is worth noting that ADAM33 is upregulated in endothelial cells and smooth muscle cells in idiopathic pulmonary arterial hypertension, suggesting that ADAM33 might be involved in the formation of the neointima in this disease. These data imply that other pulmonary diseases, such as idiopathic pulmonary arterial hypertension, may share the same biomarkers as asthma. However, further investigations are needed to verify this assumption.

TNF-*α*, IL-17, and IFN-*γ* are key inflammatory cytokines secreted by VSMCs that are involved in vascular remodeling [21]. They are the main regulators of angiogenesis as they affect cell growth and differentiation [22]. Weng et al. have found that TNF-*α* promotes vascular endothelial cell proliferation and migration, as well as the formation of microvascular branches, by regulating the Smad1/5-NFATc1 pathway [23]. It also has been shown that IL-17A, but not IL-17F, promotes the formation of pulmonary microvascular endothelial cells *in vitro* and participates in airway remodeling in asthma [24]. Additionally, IFN-*γ* induces the apoptosis of VSMCs, downregulates the expression of matrix metalloproteinases, inhibits the degradation of the endothelial basement membrane, and promotes endothelial cell apoptosis in vascular remodeling [25]. Our data showed that the silencing of the ADAM33 gene inhibited TNF-*α* secretion and increased IFN-*γ* expression in HASMCs but did not affect the production of IL-17.

The PI3K/AKT pathway mediates the biological behavior of VSMCs in various vascular remodeling events. For example, Chen et al. have found that the PI3K/AKT pathway regulates VSMC proliferation and migration in pulmonary vascular remodeling through the degradation of extracellular matrix [26]. In addition, Zhang et al. have shown that the blockade of the PI3K/AKT pathway inhibits excessive proliferation of ASMCs and excessive production of airway blood vessels [27]. Furthermore, the downstream molecule of PI3K/AKT signaling, mTOR, regulates the formation of the actin cytoskeleton and the mobility of cells via mediating the expression and activity of Rho GTPase and protein kinase C [28]. Here, we found that ADAM33 silencing suppressed the activation of the PI3K/AKT/mTOR and Rho/ROCK pathways, indicating the involvement of these signaling pathways in ADAM33-mediated airway remodeling. FOXO1 and cyclin D1 are the downstream molecules of the PI3K/AKT pathway related to cell cycle progression and proliferation. It has been shown that the overexpression of cyclin D1 promotes cell progression from the G1 phase to the S phase [29]. Also, a high expression of PI3K upregulates cyclin D1 in aortic smooth muscle cells, while treatment with a PI3K inhibitor decreases cell proliferation and DNA synthesis [30]. Another study on VSMCs has revealed that targeted inhibition of FOXO1 and cyclin D1 induces G0/G1 phase arrest and inhibits cell proliferation [31]. Our study demonstrated that ADAM33 silencing downregulated both FOXO1 and cyclin D1 in HASMCs.

## 4. Conclusions

In summary, abnormal expression of ADAM33 regulated the migration capacity and cytokine secretion of HASMCs, probably through the PI3K/AKT/mTOR and Rho/ROCK pathways. The effect of ADAM33 silencing on cell cycle progression was related to the regulation of the downstream targets of the PI3K/AKT pathway, FOXO1 and cyclin D1. These findings suggest that targeted inhibition of ADAM33 has potential clinical significance in the treatment of airway remodeling in asthma.

## Figures and Tables

**Table 1:** Primers for qRT-PCR.

Gene	Primer sequence (5′⟶3′)		Product size (bp)
	Forward	Reverse	
ADAM33	ATAGGCGTGGTGGCTCAT	TGCGGTGTCTTGCTGTG	112
PI3K	AAGAAGTTGAACGAGTGGTTGG	GCCCTGTTTACTGCTCTCCC	192
AKT	TCCTCCTCAAGAATGATGGCA	GTGCGTTCGATGACAGTGGT	181
mTOR	GCAGATTTGCCAACTATCTTCGG	CAGCGGTAAAAGTGTCCCCTG	114
Rho	ACAGGATGCAATTTGGAGGGC	GCTCATGGGCTTACACACCA	111
ROCK	AAGTGAGGTTAGGGCGAAATG	AAGGTAGTTGATTGCCAACGAA	219
FoxO1	GGATGTGCATTCTATGGTGTACC	TTTCGGGATTGCTTATCTCAGAC	86
CyclinD1	CAATGACCCCGCACGATTTC	CATGGAGGGCGGATTGGAA	146
*β*-Actin	CATGTACGTTGCTATCCAGGC	CTCCTTAATGTCACGCACGAT	250

## Data Availability

The data that support the findings of this study are available from the corresponding author upon reasonable request.
